# Discontinuation of government subsidized HIV pre‐exposure prophylaxis in Australia: a whole‐of‐population analysis of dispensing records

**DOI:** 10.1002/jia2.26056

**Published:** 2023-01-27

**Authors:** Nicholas Andrew Medland, Doug Fraser, Benjamin R. Bavinton, Fengyi Jin, Andrew E. Grulich, Heath Paynter, Rebecca Guy, Hamish McManus

**Affiliations:** ^1^ Kirby Institute University of New South Wales Sydney New South Wales Australia; ^2^ Melbourne Sexual Health Centre Monash University Central Clinical School Melbourne Victoria Australia; ^3^ Australian Federation of AIDS Organisations Sydney New South Wales Australia

**Keywords:** discontinuation, HIV, pre‐exposure prophylaxis, prevention, dispensing, retention

## Abstract

**Introduction:**

HIV pre‐exposure prophylaxis (PrEP) has been government subsidized in Australia since April 2018 and while uptake is high among men who have sex with men, rates of discontinuation are also high. The aims of this study were to examine the impact of discontinuation on overall PrEP usage, the proportion of PrEP users who discontinue and the predictors of discontinuation.

**Methods:**

We used linked de‐identified dispensing records of all government subsidized PrEP in Australia between April 2018 and September 2021: a whole‐of‐population data set. Defining discontinuation as 180 days or more without PrEP after the final dispensed supply, we calculated the number of people who discontinued at each 6‐month interval during the study period, the proportion who had discontinued 2 years after the first supply and, using Cox regression, predictors of discontinuation.

**Results:**

Of 49,164 people dispensed PrEP (98.5% male, median age 34 years), 40.3% (19,815) had discontinued by September 2021. Within 2 years of their first supply, 11,150 (37.7%) of 29,549 PrEP users had discontinued, including 10.0% after a single dispensed supply. Large variations were observed, particularly according to prescriber characteristics: discontinuation was higher among people prescribed PrEP by low caseload (**≤**10 patients) prescribers (61.2%) than by high caseload (>100 patients) prescribers (31.1%, *p*<0.001), and by prescribers practising in areas with low estimated prevalence (<1.0%) of gay men (64.1%) than high (>5%) prevalence (36.7%, *p*<0.001). Women and younger people were more likely to discontinue, while patients receiving a higher level of government subsidy were less likely. The independent predictors of discontinuation with the greatest effect size were female sex (adjusted hazards ratio [aHR] 2.99, *p*<0.001), low estimated gay prevalence of prescriber location (aHR 1.98, *p*<0.001) and low prescriber PrEP caseload (aHR 1.79, *p*<0.001).

**Conclusions:**

There are high rates of PrEP discontinuation in Australia and some populations are at increased risk of discontinuation. Strategies are needed to support persistence on PrEP and the re‐starting of PrEP during periods of risk.

## 1 INTRODUCTION

HIV pre‐exposure prophylaxis (PrEP) is highly effective in the prevention of HIV infection [1]. High coverage of PrEP interrupts transmission networks, particularly in settings with high uptake of effective antiretroviral therapy for HIV [2, 3]. In the Australian context, men who have sex with men are specifically targeted by HIV prevention strategies [4, 5] and large reductions in HIV incidence have been observed where there has been high uptake of PrEP in those at risk [6].

From 2016, PrEP was available in Australia through implementation projects which included 18,000 individuals [6, 7]. In April 2018, daily PrEP was subsidized by the Australian Government Pharmaceutical Benefits Scheme providing universal, inexpensive access to citizens and long‐term residents. In 2019, Australian guidelines were updated to include a recommendation for event‐based PrEP for cis‐gender men involving a minimum of four doses for each risk event [8]. PrEP can be prescribed by non‐specialist doctors without specific training or by accredited nurse practitioners in general practice, sexual health, community and hospital settings [9]. The government‐defined “general” subsidy allows Australian citizens, permanent residents and people from countries with which Australia has reciprocal healthcare agreements to purchase PrEP at a maximum co‐payment of AU$42.50 for a 1‐month supply. In addition, a “concessional” subsidy based on income, employment, health, disability and yearly pharmaceutical expenditure reduces the co‐payment to AU$6.80 or less [10].

A large‐scale implementation study of gay and bisexual men in Australia showed that 15% of participants discontinued PrEP and that discontinuation was associated with increased HIV incidence [11, 12]. A recent meta‐analysis found PrEP discontinuation of 39.5% in real‐world implementation studies, higher than in clinical trials and demonstration studies and with substantial regional variation [13]. As PrEP use expands, measuring discontinuation will be important to avoid overestimating community coverage and to monitor the effectiveness of PrEP as a public health intervention.

Australia is one of few countries with government‐held records of all dispensed subsidized PrEP. This provides a unique opportunity to measure PrEP use at a population level with a high degree of completeness. The aims of this study were to examine the impact of discontinuation on overall PrEP usage, the proportion of PrEP users who go on to discontinue and the predictors of discontinuation.

## 2 METHODS

### 2.1 Data sources and variables

From the Australian Government Department of Health, we obtained de‐identified linked data for all dispensed subsidized PrEP prescriptions between 1 April 2018 and 30 September 2021. Each record contains an anonymized patient code (linking prescriptions in the same patient), an anonymized prescriber code (linking prescriptions from the same prescriber), date and quantity supplied, patient age, sex and postcode, prescriber postcode and subsidy type.

### 2.2 Outcomes

We equated one dispensed tablet to 1 day of PrEP use. We defined discontinuation as more than 180 consecutive days without PrEP after the final dispensed supply without resumption before the end of the observation period. For example, if the final supply was 30 tablets, the definition of discontinuation was met 210 days after that final supply if there was no subsequent supply. We accounted for any unused previously supplied PrEP to avoid over‐estimating discontinuation in those who might have accumulated previously dispensed medicine [14].

We used a long definition of discontinuation to minimize the misclassification of event‐based PrEP users. Because dispensing records cannot determine the reason why people have not returned for more PrEP, our definition of discontinuation could include some people who use event‐driven PrEP infrequently—specifically those who used PrEP once every 30 days or less (seven or fewer risk events over the 210 days after their final supply, which would mean up to 28 tablets), but rising to once every 12 days or less in those who received 90 tablets at their final supply (22 risk events, or up to 88 tablets over 270 days).

### 2.3 Analyses

We performed two analyses of PrEP dispensing records during the 3^1/2^ years after the introduction of subsidized PrEP in April 2018. The aim of the first analysis was to quantify the impact of discontinuation on overall PrEP usage. Every 6 months from April 2018 to September 2021, we determined the number of people who met the definition of discontinuation and the number who did not (including those who had newly initiated PrEP during those 6 months and, therefore, could not have met the definition).

The aim of the second analysis was to quantify the proportion of all users who discontinued and the predictors of discontinuation. For all people who were dispensed PrEP for the first time between 1 April 2018 until 30 September 2019, we determined the proportion who had discontinued PrEP by 2 years after they were first dispensed it and compared proportions between groups defined below using Pearson's chi‐squared tests.

We included the following variables and defined comparator groups as follows: age group at first prescription (18–29 years, 30–39 years, 40 years and greater), sex recorded at first prescription (male or female), subsidy type (“concession” if concessional benefit applied at any dispensing during the study period, or “general” if not [10]), year of first supply (2018 or 2019), postcode of patient residence and postcode of prescriber practice categorized according to the published estimate of the proportion of gay men in that postal district (low <1.0%, medium 1–5.0% or high > 5% [15]), and PrEP caseload defined as the number of individual patients prescribed PrEP by each prescriber (1–10 patients, 11–100 patients or more than 100 patients). Prescriber location and PrEP caseload were assigned to each of that prescriber's patients. When the patient had more than one prescriber, the higher caseload and the higher gay prevalence area of practice was assigned. This categorization of location was chosen because communities of gay men and their health services are concentrated in certain areas and PrEP promotion strategies in Australia target men who have sex with men [4, 5, 16].

Univariable and multivariable Cox proportional hazards regression analyses were performed using time from onset of risk of discontinuation to censor (2 years) or event (when the definition of discontinuation was met) using the covariates above. To avoid immortal‐time bias, the onset of risk commenced at 180 days after the date of the first supply plus the number of tablets in the first dispensed supply, before which the definition of discontinuation cannot be met. Covariates with a *p*‐value ≤0.20 were included in the multivariable analysis. Kaplan–Meier survival curves were generated.

We performed a sensitivity analysis to examine the effect of misclassification of infrequent users of event‐based PrEP by repeating the second analysis after excluding those whose final supply was greater than 30 tablets. Infrequent users of event‐based PrEP dispensed a larger quantity would have longer periods without requiring subsequent supply and, therefore, more likely to be misclassified as having discontinued.

We also performed an analysis to examine the effect of excluding those with more than 180 days without PrEP who resumed PrEP before the end of the study period. We examined the predictors of resumption during 2 years of follow‐up in those who had met the discontinuation definition at any point during the study. We performed univariable and multivariable Cox proportional hazards regression using the same covariates and the time to resumption of PrEP (defined as the number of days from when the definition of discontinuation was met to the date of subsequent dispensed supply [event] or 2 years after the date of first supply [censor]). Covariates with a *p*‐value ≤0.20 were included from the multivariable analysis.

This study was approved by the UNSW Sydney Human Research Ethics Committee (HC190682). A waiver of consent was granted as part of this approval.

## 3 RESULTS

Between April 2018 and September 2021, 49,164 people (98.5% male) were dispensed PrEP through the Pharmaceutical Benefits Scheme in Australia. The median age was 34 years at first supply (IQR 27–43) and 19.5% had received at least one concessional subsidy. At the time of their first supply, 85.6% resided in Australia's five largest cities; and 29.5% resided in high gay prevalence areas (Table 1A). There were 14,737 prescribers, of whom 88.4% had prescribed 10 PrEP patients or fewer, 1.7% had prescribed more than 100 patients and 17.7% practised in high gay prevalence areas (Table 1B).

**Table 1:** People dispensed and prescribers of Australian government subsidized PrEP between April 2018 and September 2021

A: People dispensed PrEP		
**Total**	49,164	
Female, *n* (%)	890	(1.8%)
Age, mdn (IQR)^a^	34	(27–43)
Concessional subsidy, *n* (%)^b^	9585	(19.5%)
Follow‐up, months (IQR)	28.3	(16.0–26.1)
Days on PrEP, mdn (IQR)^c^	270	(90–630)
**Resident location of patients** ^a^		
Area with gay prevalence which is estimated as^d^:		
Low	15,799	(32.7%)
Medium	18,465	(38.2%)
High	14,096	(29.5%)
Australian state or territory		
NSW	19,266	(39.2%)
Vic	14,222	(29.0%)
QLD	8150	(16.6%)
SA	2133	(4.3%)
WA	3299	(6.7%)
ACT	1163	(2.4%)
NT	320	(0.6%)
Tas	601	(1.2%)
Urban location		
Major cities	42.047	(85.6%)
Inner regional	4864	(9.9%)
Outer Regional	1980	(4.0%)
Remote, very remote	247	(0.5%)
**Total**	14,737	
Number of prescribers with a patient caseload of^e^:		
1–10	13,025	(88.4%)
11–100	936	(6.6%)
> 100	776	(1.7%)
Range 1–1931 patients per prescriber		
Number of prescribers practising in area with gay prevalence which is estimated as:^d^		
Low	5634	(40.0%)
Medium	5979	(42.4%)
High	2491	(17.7%)
**Patients of prescribers**		
Patients of a prescriber whose PrEP caseload is^e^, ^f^:		
1–10	8205	(16.7%)
11–100	9216	(18.8%)
> 100	31,473	(64.6%)
Patients with prescriber practising in district with gay prevalence which is estimated as^d^, ^f^:		
Low	5564	(11.6%)
Medium	13,104	(27.4%)
High	29,235	(61.0%)

^a^ At the time of the first PrEP supply.

^b^ Any PrEP supply during the study period with higher concessional subsidy.

^c^ One dispensed tablet of PrEP equals 1 day on PrEP.

^d^ Estimated prevalence of gay men in area of patient residence and prescriber practice: low <1.0%, medium 1–5.0% and high >5%.

^e^ Number of individual patients with prescriptions dispensed from each prescriber.

^f^ Where one individual has been dispensed prescriptions from more than one prescriber, the higher caseload and the higher gay prevalence practice location is assigned.

**Figure 1:**
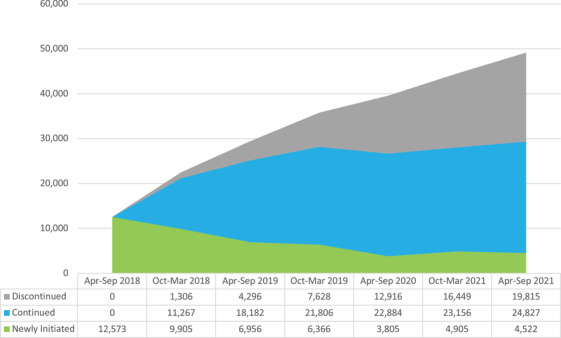
The number of people who had discontinued PrEP for more than 180 days, first received PrEP, or continued between March 2018 and September 2021.

In the first analysis, by the end of the study period, 40.3% (19,815) of all individuals who have been prescribed government subsidized PrEP had discontinued PrEP (Figure 1).

In the second analysis, we included 29,549 people who were supplied PrEP between April 2018 and September 2019. By 2 years after their first supply, 11,150 people (37.7%) had met the definition of discontinuation with more than 180 days without subsequent supply including 10.0% who had discontinued after a single dispensed supply. Those who had discontinued had a median of 537 days without PrEP (IQR 349–640) by the end of the study (Table 2).

**Table 2:** Dosing and dispensing characteristics of people who had discontinued PrEP for more than 180 days at 2 years after first supply

(*n* = 29,549^a^)	*n*	%
Discontinuation, *n* (%)	11,150	37.7%
Early discontinuation^b^ *n* (%)	2958	10.0%
Days without PrEP after final dispensed supply, mdn (IQR)	537	(349–640)
Prescriptions dispensed, mdn (IQR)	3	(1–6)
Doses supplied, mdn (IQR)	120	(60–270)
Final quantity dispensed		
< = 30	7124	63.9%
> = 90	3392	30.4%

^a^ People who initiated between April 2018 and September 2019 with all data censored at 2 years from the date of each individual's first dispensed prescription.

^b^ People who discontinued after a single dispensed supply.

After 2 years of follow‐up, the proportion who had discontinued for more than 180 days was higher in younger age groups (48.9% in under 30 years vs. 31.7% in 40 years and over, *p*<0.001), women (78.6% women vs. 37.7% men, *p*<0.001), lower gay prevalence area of residence (44.3% in low [<1.0%] prevalence vs. 31.1% in high [>5%] prevalence areas, *p*<0.001), lower gay prevalence area of prescriber practice (64.1% in low vs. 36.7% in high, *p*<0.001) and lower prescriber PrEP caseload (61.2% with a caseload of 10 or less vs. 31.1% caseload more than 100, *p*<0.001). People who initiated PrEP in 2019 (compared to 2018) were more likely to discontinue (48.2% vs. 34.3%, *p*<0.001) but people who had received a concessional subsidy were less likely (35.7% vs. 38.2%, *p*<0.001) (Table 3).

**Table 3:** The proportion of people who had discontinued PrEP 2 years after the first supply, and crude and adjusted hazards ratios for discontinuation (Cox regression analysis)

PrEP users^a^		Discontinued at 2 years^b^			Discontinuation (Cox regression)^c^					
		%	*n*	*p*‐value^d^	Unadjusted HR (CI)	*p*‐value		Adjusted HR (CI)	*p*‐value	
**PrEP user characteristics**										
Sex										
Male	29,169	37.7%	(10,853)	<0.001	ref			ref		
Female	380	78.6%	(297)		3.65	(3.25–4.09)	<0.001	2.99	(2.65–3.38)	<0.001
Age group										
18–29	8302	48.9%	(4054)	<0.001	1.76	(1.68–1.84)	<0.001	1.62	(1.55–1.70)	<0.001
30–39	10,124	35.3%	(3.566)		1.15	(1.09–1.20)	<0.001	1.15	(1.10–1.21)	<0.001
40+	11,123	31.7%	(3530)		ref			ref		
Gay prevalence area^e^										
Low	8529	44.3%	(3770)	<0.001	1.54	(1.48–1.62)	<0.001	1.02	(0.96–1.07)	0.59
Med	10,745	38.7%	(4167)		1.32	(1.26–1.39)	<0.001	1.03	(0.98–1.08)	0.29
High	9754	31.1%	(3.026)		ref			ref		
Subsidy type^f^										
General	24,368	38.2%	(9302)	<0.001	ref			ref		
Concessional	5181	35.7%	(1848)		0.90	(0.86–0.95)	<0.001	0.75	(0.72–0.79)	<0.001
Year first dispensed										
2018	22,482	34.3%	(7705)	<0.001	ref			ref		
2019	7057	48.2%	(3445)		1.60	(1.54–1.67)	<0.001	1.38	(1.32–1.44)	<0.001
Final dispensed supply										
30 tablets or less	17,188	41.5%	(7124)	<0.001	ref			ref		
More than 30 tablets	12,361	32.6%	(4025)		0.82	(0.79–0.85)	<0.001	1.09	(1.04–1.13)	<0.001
**Prescriber characteristics**										
Gay prevalence area^e^, ^g^										
Low	2585	64.1%	(1657)	<0.001	2.80	(2.65–2.96)	<0.001	1.98	(1.85–2.12)	<0.001
Med	7228	45.0%	(3252)		1.60	(1.53–1.67)	<0.001	1.37	(1.31–1.44)	<0.001
High	19,409	36.7%	(5956)		ref			ref		
Prescriber caseload^g^, ^h^										
1–10	4022	61.2%	(2463)	<0.001	2.51	(2.40–2.64)	<0.001	1.78	(1.69–1.89)	<0.001
11–100	4785	46.7%	(2234)		1.65	(1.57–1.73)	<0.001	1.48	(1.41–1.56)	<0.001
>100	20,742	31.1%	(6453)		ref			ref		

Abbreviations: CI, confidence interval; HR, hazards ratio.

^a^ People with first PrEP supply between April 2018 and September 2019 with all data censored at 2 years from the date of first supply.

^b^ Discontinuation is defined as more than 180 days without PrEP after the final dispensed supply at 2 years, equating one dose of dispensed PrEP to 1 day supply.

^c^ Time from when the definition of discontinuation could be first met until the definition was met or 2 years (censor).

^d^ Pearson's chi‐squared test.

^e^ Estimated prevalence of gay men in area of patient residence (at the time of first prescription) or prescriber practice (low <1.0%, medium 1–5.0% and high >5%).

^f^ Concessional subsidy is a higher level of government subsidy resulting in a lower patient co‐payment based on income, employment, health, health expenditure or disability.

^g^ Where one individual has been dispensed prescriptions from more than one prescriber, the higher caseload and the higher gay prevalence practice location is assigned.

^h^ Number of individuals for whom that patient's prescriber has prescribed PrEP.

**Figure 2:**
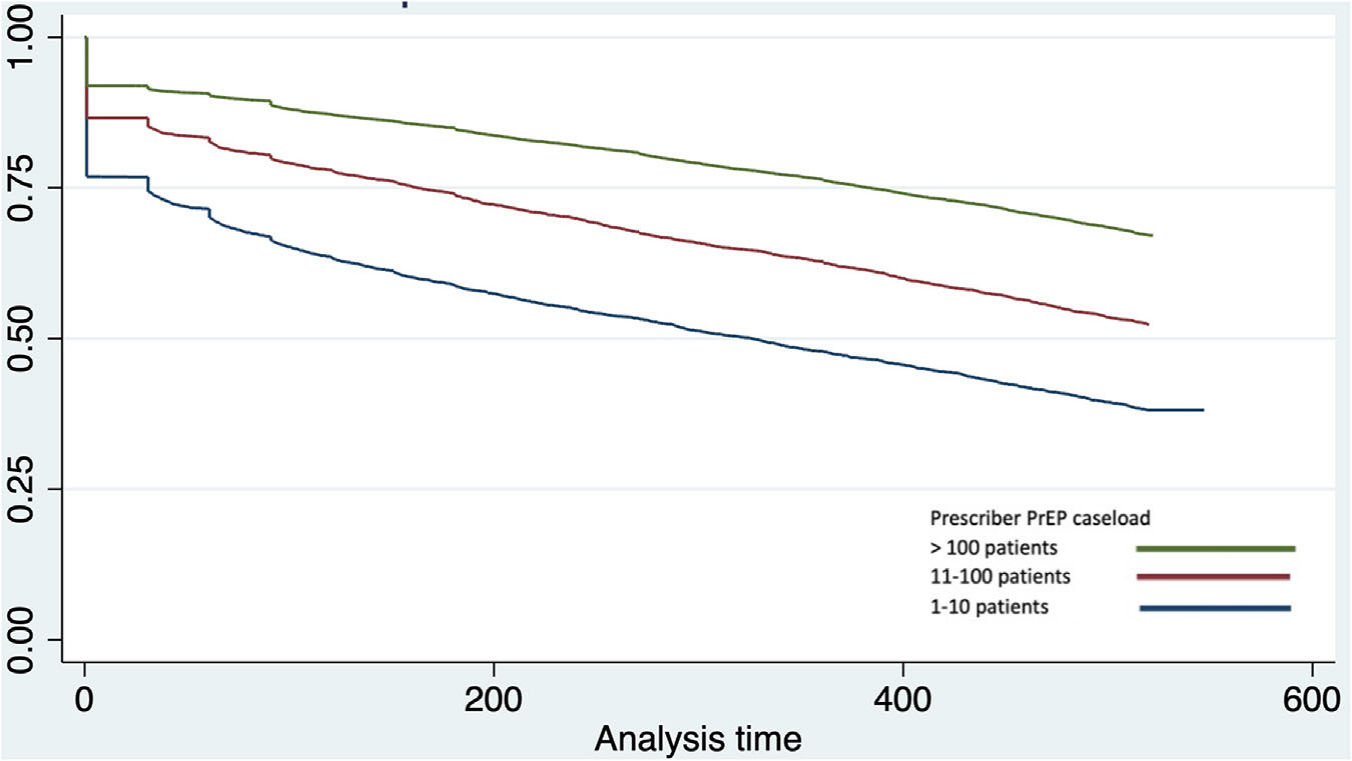
Kaplan–Meier curves for survival without discontinuation after initiating PrEP, by the caseload of the individual's prescriber.

In the Cox regression analysis, the predictors of discontinuation in both univariable and multivariable analyses were younger age group, female sex, not receiving a concessional subsidy, initiating PrEP in 2019, prescriber practice in low gay prevalence area and low prescriber caseload. Gay prevalence of patient area of residence was significant only in the univariable analysis. Sex, prescriber practice location and prescriber caseload were the predictors with the largest effect sizes (Table 3). Kaplan–Meier curves for survival without discontinuation by prescriber caseload are shown (Figure 2).

Findings were unchanged in the sensitivity analysis after excluding people with more than 30 tablets dispensed at the final PrEP supply (Table S1). In the additional analysis of PrEP resumption in those who met the definition of discontinuation at any time during the study period, the predictors of resumption were opposite in direction and effect to the predictors of discontinuation in the main analysis (Table S2).

## 4 DISCUSSION

In this study of all dispensed subsidized PrEP in Australia, 40.3%(19,815) of 49,164 people who received PrEP had discontinued for 180 days or more. By 2 years after their first PrEP supply, 37.7% of patients were no longer taking it and one in 10 received it only once. Prescriber characteristics were associated with the largest differences in discontinuation: 61.2% of patients of low caseload prescribers (1–10 patients) compared to 31.1% of high caseload prescribers (>100 patients); 64.1% of patients of prescribers practising in areas with a low estimated prevalence of gay men (<1.0%) compared to 36.7% of those whose prescriber practices in an area with high gay prevalence (>5%). Predictors of discontinuation also included being female and younger. Patients who had received a concessional government subsidy were less likely to discontinue.

PrEP discontinuation in Australia is comparable to that observed in implementation studies globally, 39.5% in a recent meta‐analysis [13]. The high rate of discontinuation makes setting targets and monitoring progress challenging: Australia has committed to a 2022 national target of 75% use in those at greatest risk of HIV acquisition [5, 17]. Meeting such targets will require an understanding of whether those who discontinued PrEP are at risk of HIV and, potentially, measures to improve persistence on PrEP.

Although some discontinue PrEP because they no longer need it, PrEP discontinuation may leave others at risk of HIV. In‐depth interviews of men who have sex with men who stopped PrEP have shown that although most did so because they felt they no longer needed it, some reported subsequent risk without PrEP which they had not predicted [18]. An implementation study conducted in Australia showed that HIV incidence was 12 times higher in those with lower persistence or adherence [11].

Large gay‐friendly clinics, gay community health promotion organizations and publicly funded sexual health clinics are a prominent feature of the Australian HIV response [16]. Australian policy is to promote and deliver PrEP through these services [5, 17].

Consistent with this, we observed that 64.6% of patients were prescribed PrEP by a doctor with more than 100 PrEP patients and 61.0% of patients by doctors practising in locations with a high prevalence of gay men. We also observed the lowest rates of discontinuation in these patients. While our study indicates that this service delivery model appears to work well for the majority who access it, it is important to understand the reasons for discontinuation in those who do not.

Quality of care could explain differences in discontinuation associated with caseload. A qualitative study of Australian PrEP prescribers reported concerns that small patient numbers made it too difficult to develop PrEP expertise and that general practitioners with low PrEP knowledge may deliver low‐quality care [9]. Stigma and discrimination related to sexuality and sexual behaviour have been identified as affecting the perception, uptake and experience of PrEP use [19]. People attending centres experienced in and oriented towards PrEP delivery may be exposed to a range of supportive health promotion messages and services protective against discontinuation.

Successful navigation of health systems to attend services supportive of PrEP may also be a marker of patient characteristics in turn associated with lower discontinuation. These characteristics might include health literacy, motivation, community connectedness and personal resources [20]. Higher engagement with gay men is associated with higher use of PrEP use in this population, a factor which could not be directly measured in our study and may also be correlated with the location of residence or location of prescriber [20].

Residents in higher gay prevalence locations were less likely to discontinue PrEP but this was not an independent predictor of discontinuation. Although only 29.5% of PrEP users lived in a high gay prevalence area, 61.0% had a prescriber practising in a high gay prevalence area and 64.6% had a prescriber with a caseload greater than 100. High gay prevalence areas are also expensive inner urban suburbs [15]. Many gay community‐engaged individuals who attend gay‐oriented health services in these areas may be unable to or prefer not to live there.

HIV prevention policy has focussed on PrEP initiation, while determinants of discontinuation remain poorly understood [21]. There is a paucity of research evaluating strategies to improve persistence [21, 22, 23]. Clinicians receive little guidance on this aspect of care. For example, although the 96‐page Australian guidelines recommend patients taking PrEP are reviewed every 3 months, they do not cover strategies to assess or sustain persistence [8].

The Australian model is to allow prescribing by prescribers without special training and without restriction on the setting. In this study, 88.4% of prescribers had 10 PrEP patients or less. This high number may partly be explained by this lack of restriction. For example, a patient may request that a low caseload doctor renew their prescription that was initiated in a high caseload setting. The future success of this model will require an understanding of the reasons that patients attending low caseload settings discontinue so frequently and ensuring that they receive adequate support and quality of care.

A published analysis of data from selected general practices also found that younger men who have sex with men were more likely to discontinue, but those receiving concessional subsidy were less likely [24]. Younger age has previously been observed to be associated with lower persistence on PrEP, and indeed with other longer‐term medications [14, 25]. We also found that the almost one in five people who received a concessional subsidy (based on income, employment, health, expenditure on medicines or disability) were at lower risk of discontinuation, despite financial barriers being identified as a predictor of discontinuation elsewhere [26]. This is an encouraging finding, demonstrating the strength of Australia's universal healthcare system.

In Australia, although more than 10% of HIV notifications are in women [27], very few women used PrEP and fewer continued it. Low uptake (and low persistence) in women may reflect difficulties in identifying women at ongoing risk of HIV. Other high‐income countries also report barriers to PrEP usage in women at risk of HIV [28, 29]. Although there is little research on PrEP discontinuation in women in high‐income settings, the high proportion who discontinue may reflect appropriate and on‐guideline use of PrEP in those situations where the risk in women is identified: during pregnancy or while a male partner initiates antiretroviral therapy [8].

We also found that those who first dispensed PrEP in 2019 were more likely to discontinue compared to 2018. Australia's large PrEP implementation studies ended and transitioned their participants to government subsidized PrEP soon after the subsidy began [6]. It is likely that a high proportion of those first receiving subsided PrEP in 2018 were participants of implementation studies, which generally report lower discontinuation [13]. In addition to higher levels of support during the trial, study participants may be more motivated or have higher baseline risk due to stricter eligibility requirements. This observation also suggests that COVID‐19 lockdowns may not substantially affect our results. For those PrEP users initiating in 2018, the 2 year study period ended in 2020 when the lockdowns were at their height and reductions in PrEP usage have previously been observed [30].

There are limitations to consider when interpreting our findings. Although the data include almost all PrEP dispensed in Australia, there are few variables available. This means that complex correlations between PrEP caseload, patient location and prescriber location which are captured in our data set and between patient or prescriber characteristics which are not must be investigated through other means. In addition, death and migrant or minority ethnic status are not recorded. Sex is recorded only as male or female so gender diversity cannot be ascertained.

Dispensing data are unable to completely distinguish between those who have discontinued and those who may be continuing to use PrEP infrequently. Community surveys have found that 17.2% of men who have sex with men taking PrEP reported using event‐based dosing [31]. We chose a conservative definition of discontinuation to minimize the misclassification of event‐based users who use PrEP infrequently. One PrEP implementation study in Australia found that participants reported sexual activity most weeks (75%) and condomless sex in 60% of weeks, thus any misclassification would be low [32]. We also performed a sensitivity analysis excluding those with a larger final supply who would be more likely to be misclassified and the findings were the same.

The data set does not include people ineligible for subsidized PrEP or who personally legally import PrEP. The former group have been identified as a priority population with high HIV vulnerability [33]. For the latter, personal importation may be a cheaper if more complicated method, and has been used by 9.7% of PrEP users according to one survey [9, 31]. People moving from subsidized to personal importation may also be miscategorized as discontinuing.

## 5 CONCLUSIONS

PrEP discontinuation in Australia is high, and one in 10 people discontinue PrEP after a single supply. Research is required to determine if people who discontinue PrEP are at risk of HIV. Association with prescriber factors suggests that quality of care may be an important factor in persistence. Clinical guidance may need to focus on the assessment of the ongoing need to ensure that usage aligns with risk. Australia, and similar countries, have set high strategic PrEP targets to eliminate HIV transmission and unless discontinuation is monitored and better understood, coverage may be over‐estimated and targets missed.

We identified groups of individuals at increased risk of PrEP discontinuation: young people, women and patients of prescribers with low PrEP caseload and outside high gay prevalence areas. It is, therefore, a priority to determine strategies to support the maintenance of PrEP during changing periods of perceived risk, and re‐starting PrEP before engaging in sexual risk behaviour.

## COMPETING INTERESTS

The authors report no potential competing interests.

## AUTHORS’ CONTRIBUTIONS

NAM performed the research, analysed the data and wrote the paper. BRB, DF, FJ, AEG, HP, RG and HM assisted with the methodology/design of the study, interpretation of the results and writing and editing of the manuscript. RG and HM supervised the first author and assisted with the statistical analysis.

## FUNDING

No specific funding was sought for this study. NAM is supported by an Australian National Health and Medical Research Council Fellowship Grant (APP1158035).

## Supporting information

Supporting information

## Data Availability

Aggregate Pharmaceutical Benefits Scheme (PBS) claims data are publicly available at https://www.pbs.gov.au/info/statistics/dos‐and‐dop/dos‐and‐dop. The PBS data in this study were used under licence from the Australian Government Services Australia. Access to these data by other individuals or authorities is not permitted without the express permission of the approving human research ethics committees and data custodians.

## References

[1] Grant RM , Lama JR , Anderson PL , McMahan V , Liu AY , Vargas L , et al. Preexposure chemoprophylaxis for HIV prevention in men who have sex with men. N Engl J Med. 2010; 363(27):2587–99. 21091279 10.1056/NEJMoa1011205 PMC3079639

[2] Weiss KM , Prasad P , Sanchez T , Goodreau SM , Jenness SM . Association between HIV PrEP indications and use in a national sexual network study of US men who have sex with men. J Int AIDS Soc. 2021; 24(10):e25826. 34605174 10.1002/jia2.25826 PMC8488229

[3] Rodger AJ , Cambiano V , Bruun T , Vernazza P , Collins S , van Lunzen J , et al. Sexual activity without condoms and risk of HIV transmission in serodifferent couples when the HIV‐positive partner is using suppressive antiretroviral therapy. JAMA. 2016;316(2):171–81. 27404185 10.1001/jama.2016.5148

[4] Health Network . HIV Strategy 2021–2015. Sydney; 2020.

[5] Australian Government Department of Health . Eighth National HIV Strategy 2018–2020. 2018.

[6] Grulich AE , Guy R , Amin J , Jin F , Selvey C , Holden J , et al. Population‐level effectiveness of rapid, targeted, high‐coverage roll‐out of HIV pre‐exposure prophylaxis in men who have sex with men: the EPIC‐NSW prospective cohort study. Lancet HIV. 2018;5(11):e629–e637. 30343026 10.1016/S2352-3018(18)30215-7

[7] UNSW Sydney . Monitoring HIV pre‐exposure prophylaxis uptake in Australia. Sydney: Kirby Institute; 2022.

[8] The Australasian Society for HIV, Viral Hepatitis and Sexual Health Medicine (ASHM) . National PrEP guidelines update: prevent HIV by prescribing PrEP. Sydney; 2021.

[9] Smith AKJ , Haire B , Newman CE , Holt M. Challenges of providing HIV pre‐exposure prophylaxis across Australian clinics: qualitative insights of clinicians. Sex Health. 2021; 18(2):187–94. 33906719 10.1071/SH20208

[10] Australian Government Department of Health and Aged Care . The Pharmaceutical Benefits Scheme. 2022. Available from: https://www.pbs.gov.au/info/about‐the‐pbs. Accessed 1 Dec 2022.

[11] Grulich AE , Jin F , Bavinton BR , Yeung B , Hammoud MA , Amin J , et al. Long‐term protection from HIV infection with oral HIV pre‐exposure prophylaxis in gay and bisexual men: findings from the expanded and extended EPIC‐NSW prospective implementation study. Lancet HIV. 2021; 8(8):e486–94. 34217426 10.1016/S2352-3018(21)00074-6

[12] Jin F , Amin J , Guy R , Vaccher S , Selvey C , Zablotska I , et al. Adherence to daily HIV pre‐exposure prophylaxis in a large‐scale implementation study in New South Wales, Australia. AIDS. 2021; 35(12):1987–96. 34101630 10.1097/QAD.0000000000002970

[13] Zhang J , Li C , Xu J , Hu Z , Rutstein SE , Tucker JD , et al. Discontinuation, suboptimal adherence, and reinitiation of oral HIV pre‐exposure prophylaxis: a global systematic review and meta‐analysis. Lancet HIV. 2022; 9(4):e254–68. 35364026 10.1016/S2352-3018(22)00030-3 PMC9124596

[14] van Epps P , Maier M , Lund B , Howren MB , Beck B , Beste L , et al. Medication adherence in a nationwide cohort of veterans initiating pre‐exposure prophylaxis (PrEP) to prevent HIV infection. J Acquir Immune Defic Syndr. 2018; 77(3):272–8. 29210835 10.1097/QAI.0000000000001598

[15] Callander D , Mooney‐Somers J , Keen P , Guy R , Duck T , Bavinton BR , et al. Australian ‘gayborhoods’ and ‘lesborhoods’: a new method for estimating the number and prevalence of adult gay men and lesbian women living in each Australian postcode. Int J Geogr Inf Sci. 2020; 34:2160–76.

[16] Pell C , Donohoe S , Conway D. Health care services for men who have sex with men in different Australian states and territories since the emergence of HIV. Sex Health. 2008; 5(2):161–8. 18588781 10.1071/sh07101

[17] NSW. HIV Strategy 2021–2025 Quarter 4 and Annual 2021 Data Report 2021. Sydney: NSW Health; 2022.

[18] Philpot SP , Murphy D , Fraser DBRB . Contexts of condomless anal intercourse after discontinuing and before recommecing PrEP among gay and bisexual men in Australia. Australiasian HIV/AIDS Conference. 2021.

[19] Rosengren AL , Lelutiu‐Weinberger C , Woodhouse EW , Sandanapitchai P , Hightow‐Weidman LB. A scoping review of HIV pre‐exposure prophylaxis stigma and implications for stigma‐reduction interventions for men and transwomen who have sex with men. AIDS Behav. 2021; 25(7):2054–70. 33389319 10.1007/s10461-020-03135-2 PMC10539076

[20] Hammoud MA , Vaccher S , Jin F , Bourne A , Maher L , Holt M , et al. HIV pre‐exposure prophylaxis (PrEP) uptake among gay and bisexual men in Australia and factors associated with the nonuse of PrEP among eligible men: results from a prospective cohort study. J Acquir Immune Defic Syndr. 2019; 81(3):e73–84. 30973548 10.1097/QAI.0000000000002047

[21] Spinelli MA , Buchbinder SP . Pre‐exposure prophylaxis persistence is a critical issue in PrEP implementation. Clin Infect Dis. 2020; 71(3):583–5. 31509603 10.1093/cid/ciz896 PMC7384308

[22] Siegler AJ , Steehler K , Sales JM , Krakower DS. A review of HIV pre‐exposure prophylaxis streamlining strategies. Curr HIV/AIDS Rep. 2020; 17(6):643–53. 32920764 10.1007/s11904-020-00528-9 PMC7671972

[23] Garrison LE , Haberer JE. Pre‐exposure prophylaxis uptake, adherence, and persistence: a narrative review of interventions in the U.S. Am J Prev Med. 2021; 61(5 Suppl 1):S73–86. 34686294 10.1016/j.amepre.2021.04.036

[24] Chidwick K , Pollack A , Busingye D , Norman S , Grulich A , Bavinton B , et al. Utilisation of pre‐exposure prophylaxis (PrEP) for HIV prevention in the Australian general practice setting: a longitudinal observational study. Sex Health. 2022; 19(2):101–11. 35469591 10.1071/SH21207

[25] Yun K , Xu JJ , Zhang J , Li JM , Hu QH , Chu ZX , et al. Female and younger subjects have lower adherence in PrEP trials: a meta‐analysis with implications for the uptake of PrEP service to prevent HIV. Sex Transm Infect. 2018; 94(3):163–8. 28756409 10.1136/sextrans-2017-053217

[26] Unger ZD , Golub SA , Borges C , Edelstein ZR , Hedberg T , Myers J . Reasons for PrEP discontinuation following navigation at sexual health clinics: interactions among systemic barriers, behavioral relevance, and medication concerns. J Acquir Immune Defic Syndr. 2022;90(3):316–24. 35286280 10.1097/QAI.0000000000002952 PMC9203912

[27] The Kirby Institute . National HIV Quarterly Report: Q1 2014–Q4 2018. Sydney: University of New South Wales; 2019.

[28] Bradley E , Forsberg K , Betts JE , DeLuca JB , Kamitani E , Porter SE , et al. Factors affecting pre‐exposure prophylaxis implementation for women in the United States: a systematic review. J Womens Health. 2019; 28(9):1272–85. 10.1089/jwh.2018.7353 31180253

[29] Nakasone SE , Young I , Estcourt CS , Calliste J , Flowers P , Ridgway J , et al. Risk perception, safer sex practices and PrEP enthusiasm: barriers and facilitators to oral HIV pre‐exposure prophylaxis in Black African and Black Caribbean women in the UK. Sex Transm Infect. 2020; 96(5):349–54. 32532928 10.1136/sextrans-2020-054457 PMC7402557

[30] Hammoud MA , Grulich A , Holt M , Maher L , Murphy D , Jin F , et al. Substantial decline in use of HIV preexposure prophylaxis following introduction of COVID‐19 physical distancing restrictions in Australia: results from a prospective observational study of gay and bisexual men. J Acquir Immune Defic Syndr. 2021; 86(1):22–30. 33027151 10.1097/QAI.0000000000002514 PMC7727320

[31] Broady T , Chan C , Bavinton B , Mao L , Molyneux A , Delhomme F , et al. Gay Community Periodic Survey. Sydney: UNSW Centre for Social Research in Health; 2021.

[32] Bavinton BR , Vaccher S , Jin F , Prestage GP , Holt M , Zablotska‐Manos IB , et al. High levels of prevention‐effective adherence to HIV PrEP: an analysis of substudy data from the EPIC‐NSW trial. J Acquir Immune Defic Syndr. 2021; 87(4):1040–7. 33852503 10.1097/QAI.0000000000002691

[33] NSW . HIV Strategy 2021–2015 Quarter 3 2021 Data Report. Sydney: NSW Department of Health; 2022.

