# Supplementary material for: Discontinuation of government subsidized HIV pre‐exposure prophylaxis in Australia: a whole‐of‐population analysis of dispensing records
Source: J Int AIDS Soc. 2023 Jan 27;26(1):e26056. doi: 10.1002/jia2.26056 (PMC9883116; doi:10.1002/jia2.26056)
Supplement: Supplementary file 1 — Supporting information [file JIA2-26-e26056-s001.docx]

**Supplementary Table 1.** **The proportion of people who had discontinued PrEP two years after first supply, and crude and adjusted hazards ratios for discontinuation (Cox regression analysis) after excluding people whose final supply was more than thirty tablets**

|  |  | **PrEP users**^1^ | **Proportion discontinued at two years^2^** | | | | | **Discontinuation (Cox regression)^3^** | | | | | | | | |  | | |  |  |
| --- | --- | --- | --- | --- | --- | --- | --- | --- | --- | --- | --- | --- | --- | --- | --- | --- | --- | --- | --- | --- | --- |
|  |  |  | % | n | *p*-value ^4^ |  | Unadjusted HR (CI) | | | *p*-value | | Adjusted HR (CI) | | | | *p*-value | |  | | |  |
|  | **PrEP user characterstics^1^** |  |  |  |  |  |  | |  | |  | |  |  |  | | | |  | | |
|  | Sex |  |  |  |  |  |  | |  | |  | |  |  |  | | | |  | | |
|  | Male | 16,889 | 40.7% | (6,879 | <.001 |  | ref | |  | |  | | ref |  |  | | | |  | | |
|  | Female | 298 | 82.2% | (245) |  |  | 3.75 | | (3.29-4.26) | | <.001 | | 3.17 | (2.77-3.62) | <.001 | | | |  | | |
|  | Age group: |  |  |  |  |  |  | |  | |  | |  |  |  | | | |  | | |
|  | 18-29 | 5,265 | 52.0% | (2,738) | <.001 |  | 1.71 | | (1.62-1.81) | | <.001 | | 1.60 | (1.52-1.70) | <.001 | | | |  | | |
|  | 30-39 | 5,551 | 38.9% | (2,153) |  |  | 1.13 | | (1.07-1.20) | | <.001 | | 1.14 | (1.07-1.21) | <.001 | | | |  | | |
|  | 40+ | 6,371 | 35.0% | (2,233) |  |  | ref | |  | |  | | ref |  |  | | | |  | | |
|  | Gay prevalence area^5^ |  |  |  |  |  |  | |  | |  | |  |  |  | | | |  | | |
|  | Low | 5,550 | 47.6% | (2,643) | <.001 |  | 1.60 | | (1.51-1.70) | | <.001 | | 1.01 | (0.94-1.08) | .80 | | | |  | | |
|  | Med | 6,529 | 42.4% | (2,766) |  |  | 1.36 | | (1.28-1.45) | | <.001 | | 1.03 | (0.96-1.10) | .46 | | | |  | | |
|  | High | 4,815 | 33.5% | (1,612) |  |  | ref | |  | |  | | ref |  |  | | | |  | | |
|  | Subsidy level^6^ |  |  |  |  |  |  | |  | |  | |  |  |  | | | |  | | |
|  | Routine subsidy | 13,744 | 42.3% | (5,817) | <.001 |  | ref | |  | |  | | ref |  |  | | | |  | | |
|  | Higher subsidy | 3,444 | 38.0% | (1,307) |  |  | 0.87 | | (0.81-0.92) | | <.001 | | 0.73 | (0.69-0.78) | <.001 | | | |  | | |
|  | Year first dispensed: |  |  |  |  |  |  | |  | |  | |  |  |  | | | |  | | |
|  | 2018 | 12,542 | 37.9% | (4,757) | <.001 |  | ref | |  | |  | | ref |  |  | | | |  | | |
|  | 2019 | 4,757 | 51.0% | (2,367) |  |  | 1.52 | | (1.44-1.59) | | <.001 | | 1.31 | (1.25-1.38) | <.001 | | | |  | | |
|  | **Prescriber characteristics** |  |  |  |  |  |  | |  | |  | |  |  |  | | | |  | | |
|  | Gay prevalence area^5,7^ |  |  |  |  |  |  | |  | |  | |  |  |  | | | |  | | |
|  | Low | 2,066 | 64.8% | (1,338) | <.001 |  | 2.77 | | (2.60-2.95) | | <.001 | | 1.95 | (1.80-2.11) | <.001 | | | |  | | |
|  | Med | 5,285 | 46.1% | (2,434) |  |  | 1.58 | | (1.50-1.67) | | <.001 | | 1.36 | (1.28-1.45) | <.001 | | | |  | | |
|  | High | 9,580 | 32.7% | (3,130) |  |  | ref | |  | |  | | ref |  |  | | | |  | | |
|  | Prescriber caseload^7,8^ |  |  |  |  |  |  | |  | |  | |  |  |  | | | |  | | |
|  | 1-10 | 3,307 | 62.3% | (2.060) | <.001 |  | 2.50 | | (2.37-2.65) | | <.001 | | 1.84 | (1.72-1.96) | <.001 | | | |  | | |
|  | 11-100 | 3,437 | 47.1% | (1,619) |  |  | 1.60 | | (1.51-1.70) | | <.001 | | 1.49 | (1.40-1.58) | <.001 | | | |  | | |
|  | >100 | 10,444 | 32.3% | (3,445) |  |  | ref | |  | |  | | ref |  |  | | | |  | | |
|  |  |  |  |  |  |  |  | |  | |  | |  |  |  | | | |  | | |

CI: confidence interval; HR: Hazards ratio: CI: confidence interval

Notes:

1. People with first PrEP supply between April 2018 and September 2019 with all data censored at 2 years from the date of each individual’s first dispensed prescription.

2. Discontinuation is defined as more than 180 days without PrEP after the final dispensed supply at two years, equating one dose of dispensed PrEP to one day supply.

3. Time from when the definition of discontinuation could be first met until the definition was met or two years (censor)

4. Pearson’s chi-squared test.

5. Estimated prevalence of gay men in area of patient residence (at the time of first prescription) or prescriber practice (low <1.0%, medium 1 to 5.0% and high >5%)

6. Concessional subsidy is a higher level of government subsidy resulting in a lower patient co-payment based on income, employment, health, health expenditure or disability

7. Where one individual has been dispensed prescriptions from more than one prescriber, the higher caseload and the higher gay prevalence practice location is assigned

8. Number of individuals for whom that patient's prescriber has prescribed PrEP

**Supplementary Table 2. Predictors of resumption in all patients with more than 180 days without PrEP (Cox regression analysis).**

|  |  |  | **Resumption (Cox regression)^1^** | | | | | |  |
| --- | --- | --- | --- | --- | --- | --- | --- | --- | --- |
|  |  |  | Unadjusted HR (CI) | | *p*-value | Adjusted HR (CI) | | *p*-value |  |
|  | **PrEP user characteristics**  Sex |  |  |  |  |  |  |  |  |
|  | Male |  | ref |  |  | ref |  |  |  |
|  | Female |  | 0.24 | (0.16-0.34) | <.001 | 0.28 | (0.19-0.41) | <.001 |  |
|  | Age group: |  |  |  |  |  |  |  |  |
|  | 18-29 |  | 0.90 | (0.83-0.98) | .013 | 0.99 | (0.91-1.08) | .85 |  |
|  | 30-39 |  | 1.10 | (1.09-1.20) | .023 | 1.12 | (1.04-1.22) | .005 |  |
|  | 40+ |  | ref |  |  | ref |  |  |  |
|  | Gay prevalence area^2^ |  |  |  |  |  |  |  |  |
|  | Low |  | 0.68 | (0.62-0.74) | <.001 | 0.93 | (0.85-1.01) | .095 |  |
|  | Med |  | 0.83 | (0.76-0.89) | <.001 | 1..01 | (0.94-1.10) | .74 |  |
|  | High |  | ref |  |  | ref |  |  |  |
|  | Subsidy level^3^ |  |  |  |  |  |  |  |  |
|  | General Subsidy |  | ref |  |  |  |  |  |  |
|  | Concessional Subsidy |  | 1.28 | (1.19-1.40) | <.001 | 1.51 | (1.39-1.65) | <.001 |  |
|  | Year first dispensed: |  |  |  |  |  |  |  |  |
|  | 2018 |  | ref |  |  | ref |  |  |  |
|  | 2019 |  | 0.91 | (0.85-0.98) | .011 | 1.00 | (0.94-1.08) | .90 |  |
|  | **Prescriber characteristics** |  |  |  |  |  |  |  |  |
|  | Gay prevalence area^2, 4^ |  |  |  |  |  |  |  |  |
|  | Low |  | 0.31 | (0.27-0.35) | <.001 | .40 | (0.35-0.47) | <.001 |  |
|  | Med |  | 0.65 | (0.60-0.70) | <.001 | .73 | (0.67-0.78) | <.001 |  |
|  | High |  | ref |  |  | ref |  |  |  |
|  | Prescriber caseload^4,5^ |  |  |  |  |  |  |  |  |
|  | 1-10 |  | 0.38 | (0.34-0.43) | <.001 | 0.52 | (0.47-0.59) | <.001 |  |
|  | 11-100 |  | 0.72 | (0.66-0.78) | <.001 | 0.74 | (0.68-0.81) | <.001 |  |
|  | >100 |  | ref |  |  | ref |  |  |  |
|  |  |  |  |  |  |  |  |  |  |

CI: confidence interval; HR: Hazards ratio: CI: confidence interval

Notes:

1. Time from when the definition of discontinuation was met to subsequent dispensed supply (event) or two years after date of first supply (censor)
2. Estimated prevalence of gay men in area of patient residence (at the time of first prescription) or prescriber practice (low <1.0%, medium 1 to 5.0% and high >5%)
3. Concessional subsidy is a higher level of government subsidy resulting in a lower patient co-payment based on income, employment, health, health expenditure or disability
4. Where one individual has been dispensed prescriptions from more than one prescriber, the higher caseload and the higher gay prevalence practice location is assigned
5. Number of individuals for whom that patient's prescriber has prescribed PrEP
